# Persistent Homology Approach for Human Presence Detection from 60 GHz OTFS Transmissions

**DOI:** 10.3390/s23042224

**Published:** 2023-02-16

**Authors:** Roman Maršálek, Radim Zedka, Erich Zöchmann, Josef Vychodil, Radek Závorka, Golsa Ghiaasi, Jiří Blumenstein

**Affiliations:** 1 Department of Radio Electronics, Brno University of Technology, 601 90 Brno, Czech Republic; 2 Silicon Austria Labs GmbH, 4040 Linz, Austria

**Keywords:** OTFS, millimeter-waves, persistent homology, CFAR, person detection

## Abstract

Orthogonal Time Frequency Space (OTFS) is a new, promising modulation waveform candidate for the next-generation integrated sensing and communication (ISaC) systems, providing environment-awareness capabilities together with high-speed wireless data communications. This paper presents the original results of OTFS-based person monitoring measurements in the 60 GHz millimeter-wave frequency band under realistic conditions, without the assumption of an integer ratio between the actual delays and Doppler shifts of the reflected components and the corresponding resolution of the OTFS grid. As the main contribution of the paper, we propose the use of the persistent homology technique as a method for processing gathered delay-Doppler responses. We highlight the advantages of the persistent homology approach over the standard constant false alarm rate target detector for selected scenarios.

## 1. Introduction

In the past ten years, there has been continuous research on replacing camera-based technology with their counterparts employing radio signal transmissions [1]. For example, Ultra Wide Band (UWB) [2] or Frequency-Modulated Continuous-Wave (FMCW) [3] signals were widely investigated to detect the presence and location of individuals or groups of persons.

The sixth generation (6G) wireless communication systems are expected to closely integrate sensing together with wireless communication. One of the key enablers for high-precision sensing is the envisaged exploitation of higher-frequency bands, either in millimeter-wave (mm-wave), sub-THz or even THz frequencies [4]. Integrated Communication and Sensing (ICaS) systems [5] have the potential to provide an alternative to dedicated environment sensing and person monitoring technologies used, e.g., for home control, elderly people fall detection, gaming, or person vital function monitoring applications. This has already been reflected in standardization activities. Recently, an IEEE 802.11bf task group has been established to create a new amendment to the IEEE 802.11 Wireless Local Area Networks (WLAN) standards family, to provide advanced sensing requirements while minimizing the effect on communications [6].

Orthogonal Frequency Division Multiplex (OFDM) is widely used in contemporary communication systems and is extensively researched in radar and target detection applications. The OFDM-based radars have been investigated, fabricated, and tested in various frequency bands and use cases, including a C-band automotive radar [7] with the results presented in the form of max-hold plots in the delay-Doppler domain. An OFDM radar with 200 MHz bandwidth and 500 MHz sampling frequency operating in the 77 GHz mm-wave band tested in the task of static and moving target detection was presented in [8].

Recently, a new communication waveform, named Orthogonal Time Frequency Space (OTFS), has been proposed [9], and has been declared to have superior performance over OFDM in doubly dispersive channels [10]. There have not been many OTFS transmission experiments so far, but some exceptions can be mentioned, such as the Software Defined Radio (SDR) based experiment [11] highlighting the problem of RF front-end impairments’ influence. These were further studied by simulation in [12]. The OTFS transmission performance in mm-waves was simulated for 28 GHz [13] or 60 GHz [14] bands. The use of OTFS scheme has also been investigated in potential radar applications, but rather through simulations, often under simplified assumptions. A comparison of OTFS and OFDM for object range and velocity estimation was presented in [15]. In [16], the OTFS-based automotive radar was analyzed, but with an assumption of delays and Doppler shifts induced by the targets are integer multiples of designed delay and Doppler grid resolution. The same assumption was set in a thorough investigation of a 28 GHz radar in [17].

Constant False Alarm Rate (CFAR) detectors [18] represent one common, robust, and practical option to detect the targets in two-dimensional (2D) data, with the noise power estimated from the cells neighboring the expected targets. Although several advanced variants of CFAR detectors with adaptive threshold settings [19] have been proposed, the CFAR detectors suffer from performance degradation in non-homogenous environments [20], and the use of CFAR may be suboptimal if the detected target spans over several bins in the delay-Doppler grid. In general, conventional CFAR detectors fail if applied to complex scenarios, especially with multiple targets, as a result of target masking [21]. In such a case, a persistent homology method [22] from the family of topological approaches [23] may be more suitable, as it does not require background noise estimation and is thus more immune to the additional targets in the vicinity of the cell under test. Moreover, it naturally assigns significance (persistence) to each of the detected targets.

Having in mind the above-mentioned state-of-the-art description, the most important contributions of this paper can be described as follows:

- We present the first-ever application of OTFS-based joint communication and sensing approach for human monitoring. We target the millimeter-wave license-exempt frequency band, and present our unique measurement test-bed for 60 GHz OTFS transmissions;
- We present persistent homology as an innovative approach for the detection of targets from two-dimensional delay-Doppler data;
- We demonstrate OTFS-based sensing with the realistic assumption of the targets representing non-integer multiples of designed delay and Doppler resolution of the OTFS system, and we compare the performance of the persistent homology detector with a conventional detector based on the constant false alarm rate.

The rest of the paper is organized as follows. In Section 2, the principle of OTFS is briefly explained, together with embedded pilots for channel estimation as well as delay and Doppler resolution. Section 3 deals with CFAR target detection and presents the principle of the persistent homology approach. In the following Section 4, the used 60 GHz measurement test-bed is described, with the results presented in Section 5. The final Section 6 rounds up the paper and presents some ideas for further work.

## 2. Orthogonal Time Frequency Space Communication

The main promise [9] behind OTFS is, under the hypothesis of the sufficient delay-Doppler resolution, to transform the time-varying multipath channel impulse response into a 2D function in the delay-Doppler domain, with each symbol experiencing the same channel gain.

### 2.1. OTFS Modulation

The OTFS information symbols (usually QAM symbols) x[k,l], placed in the delay-Doppler domain with a delay domain index k=0,1,…,N−1 and a Doppler domain index l=0,1,…,M−1, are first spread in the time-frequency domain with symbols X[n,m] through a 2D Inverse Symplectic Finite Fourier Transform (ISFFT) according to:

(1) $$X\left[n,m\right]=\mathrm{ISFFT}\left\{x[k,l]\right\}=\sum_{k=0}^{N-1}\sum_{l=0}^{M-1}x\left[k,l\right]e^{-j2\pi \left(\frac{ml}{M}-\frac{kn}{N}\right)}.$$

Note that the delay-Doppler symbols x[k,l] can be either the data symbols $x_{d}\left[k,l\right]$ or the pilot symbols $x_{p}\left[k,l\right]$. Subsequently, the signal X[n,m] is transformed from the time-frequency to the time domain using a Heisenberg transform:

(2) $$x\left(t\right)=\sum_{n=0}^{N-1}\sum_{m=0}^{M-1}X\left[n,m\right]w_{TX}\left(t-nT_{sym}\right)e^{j2\pi m\Delta f\left(t-nT_{sym}\right)},$$

where $w_{TX}$(*t*) is the transmit pulse shape, $T_{sym}$ and Δf are the symbol duration and tone separation that (inversely) define a quantization step in the delay and Doppler grid.

In general, two variants of OTFS have been studied so far. Firstly, the original one, with a single cyclic prefix (CP) before transmission of the whole N×M data symbol matrix and, secondly, its OFDM-based alternative [24] with a cyclic prefix before each of the ‘corresponding OFDM symbols’. In order not to reduce spectral efficiency, we consider the usage of a single CP.

### 2.2. Embedded Pilots for Channel Estimation

A reliable channel estimation method is needed for any of the data detection approaches. A suitable way would be to use a pilot-slots approach, where the pilot time slots were interleaved with the time slots carrying the data. In such a case, the channel state information (CSI) may become obsolete in highly time-varying channels.

**Figure 1:**
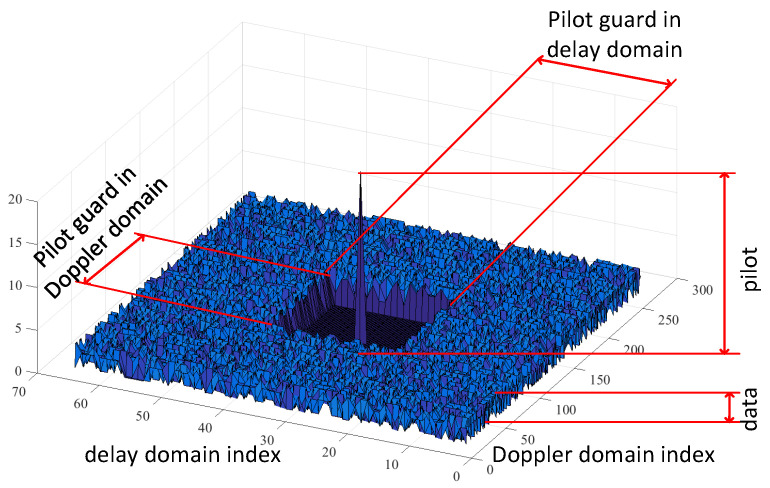
Symbols in delay-Doppler domain with the embedded pilot surrounded by the guard in both delay and Doppler domains. © 2023 IEEE. Reprinted, with permission from [14].

Thus, the use of delay-Doppler grid embedded pilots has been proposed in [25], so that the channel estimation can be performed for each OTFS slot. The principle of the embedded pilot is illustrated in Figure 1. The pilot is placed at some chosen position $k_{p},l_{p}$ in the delay-Doppler domain and is surrounded by the guard interval (zeros) in both domains. The remaining positions in the delay-Doppler grid serve for data transmission in the form of, e.g., QAM symbols.

OTFS-based communication receivers use the estimated channel state information for channel equalization. A variety of algorithms are available, ranging from iterative detection methods [26] to low-complexity approaches for OFDM-based OTFS modems [24]. As this paper targets the sensing part of the selected ICaS scenario, we will not describe the demodulation algorithms at all.

### 2.3. Delay-Doppler Resolution and Grid Mismatch

The granularity of the delay-Doppler grid with respect to real parameters of a wireless communication channel determines the OTFS communication performance in various scenarios and environments. Its setting is also crucial for sensing applications, as the delay granularity influences the ability to determine the distance to the objects, while the Doppler granularity is crucial to correctly estimating the speed of moving objects. The granularity in the delay domain $\Delta_{d}$ can be expressed as:

(3) $$\Delta_{d}=\frac{1}{M\Delta f},$$

and the granularity in the Doppler domain $\Delta_{D}$ equals

(4) $$\Delta_{D}=\frac{1}{NT_{sym}},$$

where the symbol time $T_{sym}$ is equal to the reciprocal value of subcarrier spacing Δf, and *N*, *M* are dimensions of the delay-Doppler grid.

**Figure 2:**
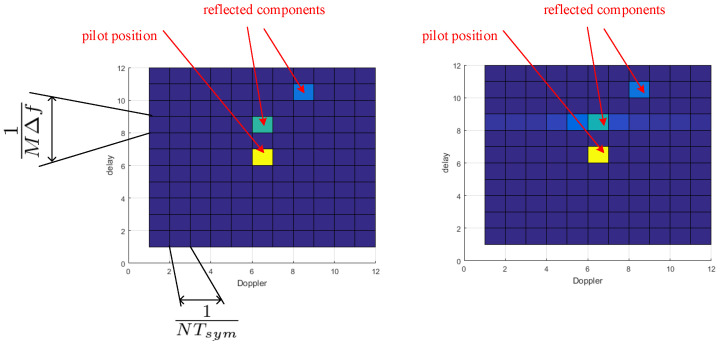
Illustration of delay-Doppler grid without (**left**) and with (**right**) grid mismatch. The leakage of one of the reflected components into the adjacent symbols is visible.

OTFS equalizers generally perform well in the case of the actual Doppler shift being an integer multiple of the Doppler-domain granularity, while in the non-integer case, special countermeasures must be taken [25]. The non-integer ratio of the actual delay and Doppler with respect to the grid resolution manifests in leakage of the multipath component over several neighboring delay-Doppler elements, as illustrated in Figure 2. In [14], we evaluated the bit error rate (BER) of OTFS in the Doppler grid mismatched case, with such mismatch defined as the remainder after dividing the actual Doppler shift by the Doppler-domain granularity $\frac{1}{NT_{sym}}$. Similar effects are also observed in the case of a non-integer multiple of the actual delay and the OTFS delay grid resolution. Note that the effect of grid mismatch has not been sufficiently considered throughout the state-of-the-art work on OTFS-based radars [16,17].

## 3. Target Detection Approaches

### 3.1. Constant False Alarm Rate Detector

As briefly stated in the introduction, CFAR represents one of the popular methods for target detection in a noisy environment [18]. During the experiments, the implementation of the cell-averaging CFAR detector from the Phased Array System Toolbox of Matlab was used. We will denote this as conventional CFAR, where details of this algorithm can be found in [27].

**Figure 3:**
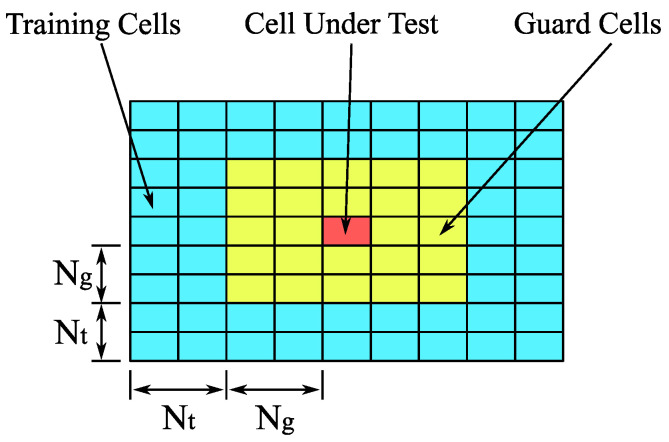
CFAR detector principle.

Figure 3 illustrates the principle of the two-dimensional CFAR detector. The basic CFAR detector estimates the noise variance $P_{N}$ as an average of the training cells and the signal variance $P_{S}$ is taken from the cell under test. The decision about the target presence is made according to the inequality

(5) $$\frac{P_{S}}{P_{N}}>\alpha ,$$

where α is the threshold, which can be calculated from the given constant alarm probability $P_{fa}$ and the number of training cells $N_{tc}$ as

(6) $$\alpha =N_{tc}\left(P_{fa}^{-1/N_{tc}}-1\right).$$

### 3.2. Persistent Homology

The problem of target identification from delay-Doppler images is equivalent to searching for local maxima in the 2D grayscale image. Persistent homology [22] belongs to the family of topological data analysis (TDA) methods, providing insights into the shape of the data [28] in a noisy environment. In principle, it is very close to the so-called watershed transform from image processing, with the name referring to a drainage divide that separates the adjacent drainage basins in a 2D grayscale image, see [29]. A tutorial on persistent homology can be found on the webpage [30], and a detailed mathematical explanation in [22].

#### Pictorial Description (without Math)

**Figure 4:**
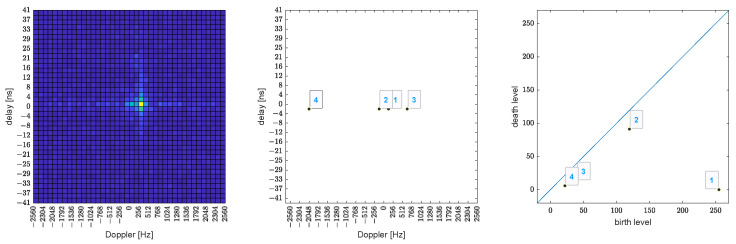
Persistent Homology example for one OTFS slot. Delay-Doppler 2D plot (**left**), identified local maxima (**middle**), and the persistence diagram (**right**). The numbers in the middle and right image denote the index of the identified peak, with index 1 corresponding to the most significant one.

Consider the delay-Doppler map as a graph of a 2D function that assigns to each pixel the height of the absolute value of the delay-Doppler bin, see the left-hand side of Figure 4. Let us now consider a fictitious water level that starts at the global maximum and continuously decreases to lower levels. Islands will form at local maxima (this action is called birth). At saddle points, two islands merge into one. We consider the lower island as merged with the higher one (death of the lower one). The so-called persistence diagram, see Figure 4 right-hand side, shows the death versus birth values (water levels) of all islands. The persistence of an island is then the difference between the birth and death levels. The further an island is from the main diagonal, the more importance (=persistence) it has.

Now the islands (peaks) can be ordered by decreasing persistence. In the delay-Doppler map, one can draw the locations of the births of the islands, see the center of Figure 4. This method not only provides the local maxima, but also quantifies their “significance” using the persistence mentioned above. If necessary, one can filter out islands with too little persistence.

## 4. Experiments

### 4.1. Measurement Test-Bed

**Figure 5:**
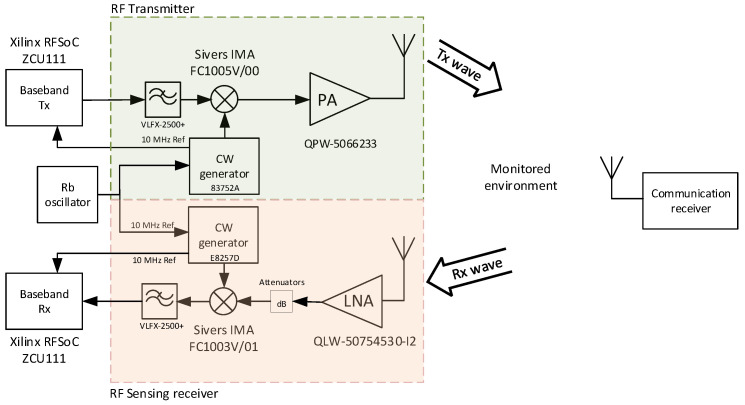
Test-bed in joint communication and sensing scenario.

Throughout the research described in this paper, we used our 60 GHz test-bed in the joint communication and sensing scenario depicted in Figure 5. The radio frequency transmitter sends the data to the receiver to whom it communicates (communication receiver), while part of the information is eventually reflected from the persons or objects located inside the monitored environment. The reflected signal is processed in the sensing receiver collocated with the transmitter. As our measurement test-bed is equipped with a single receiver only, during the experiments, we have been focusing only on the processing of the reflected signals, i.e., on the sensing task. In the following, we will thus describe the detailed structure of the transmitter and the sensing receiver only.

The baseband parts of the transmitter and the sensing receiver are based on two Xilinx Zynq UltraScale+ RFSoC ZCU111 development boards. At the transmitter, fast 4.096 GSps D/A converters (DAC’s) of the first ZCU111 board are used to generate an intermediate frequency signal with a bandwidth of up to 512 MHz. The second ZCU111 serves to sample the baseband signals at the sensing receiver with its fast A/D converters (ADCs), also clocked at 4.096 GSps. Both ZCU111 boards are controlled by the host PC via an Ethernet connection, and the received data are stored in the receiving ZCU111 M.2 SATA hard drive. The results of the measurements can be transferred to the host PC via an Ethernet connection.

**Figure 6:**
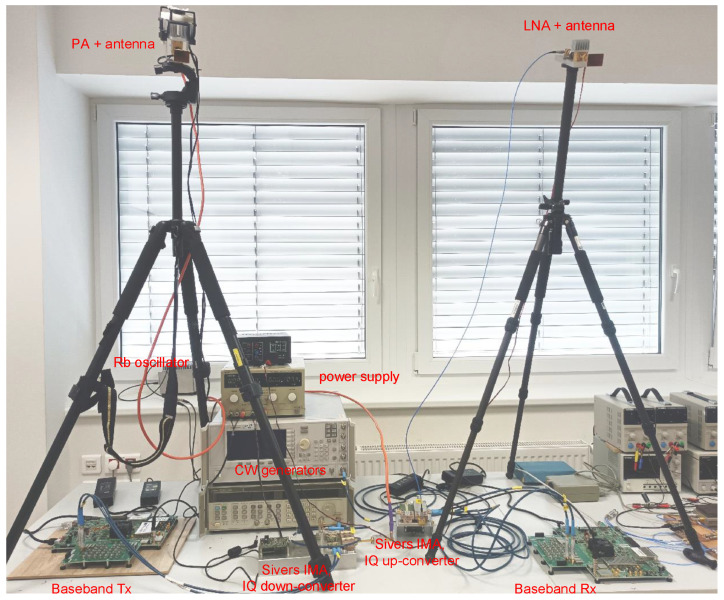
Photo of assembled test-bed with the power amplifier and low noise amplifier subsystems mounted on the tripods.

At the transmitter, the signal in the form of its I and Q components is filtered by a pair of low-pass filters VLFX-2500+, and upconverted to the desired millimeter wave frequency by the Sivers IMA FC1005V/00 up/down converter operational within the frequency range of 57–66 GHz and providing up to 5 GHz bandwidth. The Agilent 83752A generator is used to provide a low phase noise local oscillator signal with stable frequency for the upconversion. The RF signal power is boosted by the QuinStar QPW-50662330-C1 power amplifier (50–60 GHz frequency band, 30 dB gain, P1dB of 23 dBm) and transmitted using a waveguide horn antenna. At the sensing receiver, the signal reflected from the persons within the monitored area is received by a processing chain analogous to the transmitting side of the measurement test-bed. The signal is received by the same type of waveguide horn antenna and amplified by the Quinstar QLW-50754530-I2 low noise amplifier (frequency range 50–75 GHz, 30 dB gain, and the typical noise figure of 5 dB). If needed, the input level to the downconverter can be adjusted by a variable attenuator. The downconversion is performed by the Sivers IMA FC1003V/01 up/down converter with a local oscillator signal generated by the Agilent E8257D generator. After the low-pass filtering by the second pair of VLFX-2500+ filters, the baseband signal is processed in ZCU111 board. During all experiments, the ZCU111 boards and the two signal generators were synchronized by the 10 MHz reference generated by a Rubidium (Rb) oscillator. The photo of the assembled test-bed is shown in Figure 6.

**Figure 7:**
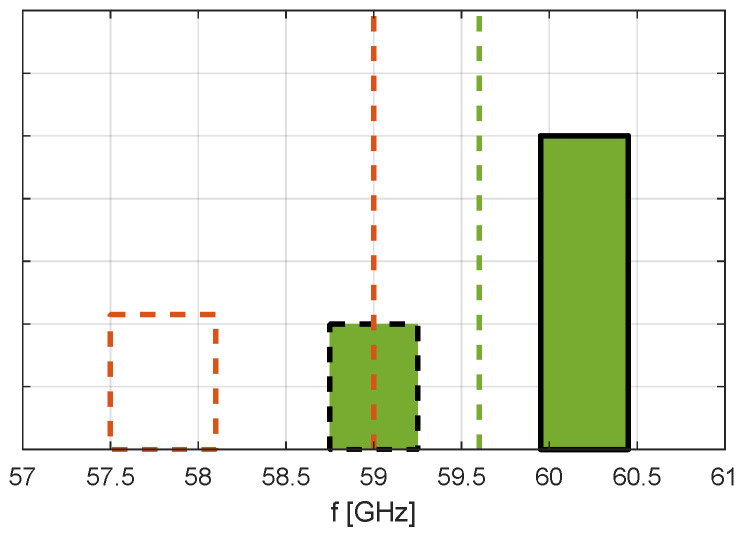
Frequency plan for up- and down-conversion. The useful signal is centered at 60.2 GHz, its partially compensated image at 59.0 GHz, the receiving image at 57.8 GHz does not influence the useful signal.

The hardware impairments of the RF front-end can seriously degrade the performance of wideband transceivers, including the OTFS millimeter wave systems [14]. Full compensation of the RF impairments is challenging, especially for wideband systems such as our 60 GHz test-bed. In order to eliminate the effects of the millimeter wave band converter’s I/Q imbalances used in our test-bed, the frequency shifting is performed as follows, with the overall frequency plan illustrated in Figure 7. The broadband complex baseband OTFS signal with a sampling frequency of 512 MHz is first digitally interpolated inside the ZCU111 board to match the DACs’ sampling frequency of 4.096 GHz. Then, it is digitally shifted by 600 MHz, the I/Q corrections are applied, and then the resulting signal is upconverted to the millimeter wave band with a center frequency of the mixer equal to 59.6 GHz. This results in a useful signal centered around 60.2 GHz (59.6 GHz + 0.6 GHz) and its (partially compensated) image centered at 59.0 GHz (green color in Figure 7).

The center frequency of the receiver mixer is set to 59.0 GHz. The downconverted signal is sampled by the ZCU111 board with a sampling frequency of 4.096 GHz, digitally shifted by −1200 MHz, filtered, and decimated to the resulting baseband sampling frequency of 512 MHz. The receiver’s I/Q imbalances could also be compensated, but as the receiving image band is centered around 57.8 GHz (59 GHz − 1.2 GHz, see the red-dotted rectangle in Figure 7), where there is no transmitted signal, this is of no concern.

### 4.2. Scenario

The OTFS wireless transmission and sensing measurements have been carried out in the laboratory environment, with the measurement test-bed placed on the table and the transmitting and receiving antennas mounted on tripods. This configuration allows easy control of the height and tilt of the antennas. The antenna position was inspired by previous work on person counting [31], with antennas mounted at a height of 2.5 m with a downward tilt of 30 degrees. The underlying idea is to sense the environment from above persons, while at the same time limiting the effects of reflections from the ground.

**Figure 8:**
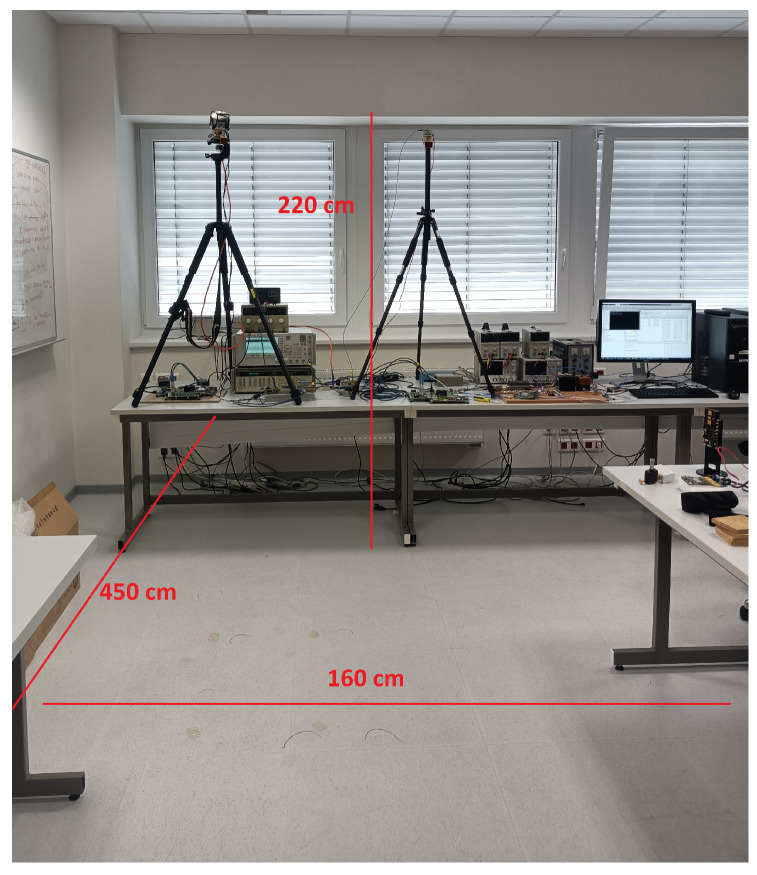
Measurement scenario with physical dimensions of the monitored environment.

As the main intention of the experiment is to demonstrate the function of the persistent homology detector in realistic conditions, we limited the investigations to the scenario of only one person present and relatively directional horn antennas; so, in ideal conditions, only one target would be detected. We have also conducted experiments with multiple persons, but since individual persons can be obscured by each other, the data measured in this way are not very suitable for validating the detector. The photo, together with a sketch of physical dimensions, is shown in Figure 8. The monitored area, in which the person can move, is limited by the laboratory tables on the left and right-hand sides.

To generate a variety of different delay and Doppler profiles of a person’s movement, we considered several simple scenarios of their presence and activity, such as:

1. A person standing still in front of antennas;
2. A person randomly walking in the monitored area;
3. A person running out from the monitored area;
4. A person in front of the antennas jumping/dancing.

### 4.3. OTFS Configuration

The parameters of the used OTFS modulation are shown in Table 1. The parameters have been tailored to the envisaged application in human monitoring and activity detection in an indoor environment. The used setting gives us a potential for approximately 60 cm resolution in position and around 1 km/h resolution in speed. For other types of environments and potential objects to detect, the parameters would need to be changed accordingly. To limit the spectrum of the transmitted signal, one hundred zero subcarriers are inserted in the frequency domain prior to the Heisenberg transform (Equation (2)).

### 4.4. Receiver Signal Processing

The received waveform from the test-bed is stored in a binary form and transferred to the control PC. Note that, with 512 MSps sampling speed, 16-bit integer format, and complex I and Q data, the data rate to store corresponds to 2 GB for every second of the experiment. Most of the subsequent processing is performed in a MATLAB environment, except for the persistent homology computation, which is a Python code obtained from [32].

The received signal is for the first time synchronized via cross-correlation of the received time-domain signal with its known replica, then fine synchronization via an FFT-based approach [33] is performed. Next, the received signal is gain-compensated for a one-tap complex attenuation factor. This makes the synchronization task simple. In real communication scenarios, some kind of synchronization preambles can be used.

Subsequently, after the cyclic prefix removal, all the OTFS frames are processed with the function inverses to Equations (1) and (2) to transform the received signal to the delay-Doppler domain. As our primary interest is on the sensing part, all the data symbols are discarded and only the guard band around the embedded pilot (see Figure 1) is further processed. Note that the size of the guard band in both delay and Doppler domains has been set to eliminate the interference between the data and the reflected pilot. If this is not the case, the modulation symbol cancellation technique from [17] should be used.

### 4.5. Persistent Homology and CFAR—Implementation and Settings

The parameters of persistent homology and CFAR detectors are summarized in Table 2. Three different approaches for target detection have been evaluated—a standard CFAR detector and two variants of the persistent homology detector technique:

1. Conventional CFAR
  The standard CFAR detector serves as a reference. The implementation of the CFAR detector from MATLAB’s Phased array toolbox was used [27]. The guard and training size parameters $N_{g}$ and $N_{t}$ have been found empirically, and they were set to the equal value in both delay and Doppler domains, see Figure 3. Their relation to $N_{tc}$ in (6) is then given by
  (7) $$N_{tc}=4N_{t}\left(1+2N_{g}+N_{t}\right).$$
  Since the output of the conventional CFAR is a map of detected targets in a form of clusters of logical ones, an additional topological algorithm was needed for counting the distinct clusters, i.e., targets. The MATLAB function bwconncomp(x, conn) with its connectivity parameter conn set to four was used for this purpose. The results are presented for two sets of $N_{g}/N_{t}$ cell sizes, i.e., 3/8 and 2/10.
2. Persistent homology applied on observations.
  In this first variant of the persistent homology detector, the method was applied directly to the estimated absolute values of the delay-Doppler map.
  For the persistent homology computations, we would like to acknowledge Prof. Huber for his persistent homology toolbox created in Python [32]. To set up a threshold for target detection, the z-scores were calculated from the potential target persistencies. The standardized z-score for the persistence of the *i*-th potential target is:
  (8) $${\text{z-score}}_{i}=\frac{{\mathrm{persistence}}_{i}-\mu }{\sigma },$$
  where μ and σ represent the mean and standard deviation of the persistencies of all potential targets. The z-score of 6 was chosen as the threshold value.
3. Persistent homology applied on SNR map.
  Before the application of the plain persistent homology approach, a Signal to Noise Ratio (SNR) for each cell from the delay-Doppler grid is computed as the ratio between the power in the cell under test and the noise variance in the training cells, similarly to in Equation (5). If we define the delay-Doppler image as a matrix H with dimensions $N_{\tau }$ in the delay domain and $N_{\nu }$ in the Doppler domain, the signal part of the SNR map matrix at coordinates m,n is defined as
  (9) $$P_{S}\left(m,n\right)=H^{2}\left(m,n\right),$$
  where H(m,n) is a single entry of H at coordinates m,n, with $n\in \langle N_{t}+N_{g},N_{\tau }-N_{t}-N_{g}-1\rangle$ and $m\in \langle N_{t}+N_{g},N_{\nu }-N_{t}-N_{g}-1\rangle$. The SNR map matrix dimension is then $N_{\tau }-2N_{t}-2N_{g}$ in the delay domain and $N_{\nu }-2N_{t}-2N_{g}$ in the Doppler domain. The noise part is defined as
  (10) $$P_{N}\left(m,n\right)=\frac{1}{N_{tc}}\sum_{k=-N_{g}-N_{t}}^{N_{g}+N_{t}}\sum_{k^{\prime }=-N_{g}-N_{t}}^{N_{g}+N_{t}}H^{2}\left(m+k,n+k^{\prime }\right)-\sum_{l=-N_{g}}^{N_{g}}\sum_{l^{\prime }=-N_{g}}^{N_{g}}H^{2}\left(m+l,n+l^{\prime }\right).$$
  The SNR map matrix entries V(m,n) are then given by
  (11) $$V\left(m,n\right)=\sqrt{\frac{P_{S}\left(m,n\right)}{P_{N}\left(m,n\right)}}.$$
  The same guard and training cell settings $N_{g}$ and $N_{t}$, as used for the reference conventional CFAR detector, were applied. Subsequently, the persistent homology method, with the same properties and settings as in the previous variant of the detector (persistent homology applied on observations), was applied to the SNR map (11).

Table 2 also quantifies the time complexity in the seconds necessary to process one delay-Doppler image by each of the investigated detectors. It was estimated on a standard personal computer with Intel(R) Core(TM) i7-2600 CPU @ 3.40GHz. Note that the complexity of the proposed persistent homology approach implementation is much higher compared to the complexity of the used CFAR detector. Nevertheless, the increased time complexity may result from calling the external Python library to implement the persistent homology.

## 5. Results

### 5.1. Delay-Doppler Plots for Selected Activities

**Figure 9:**
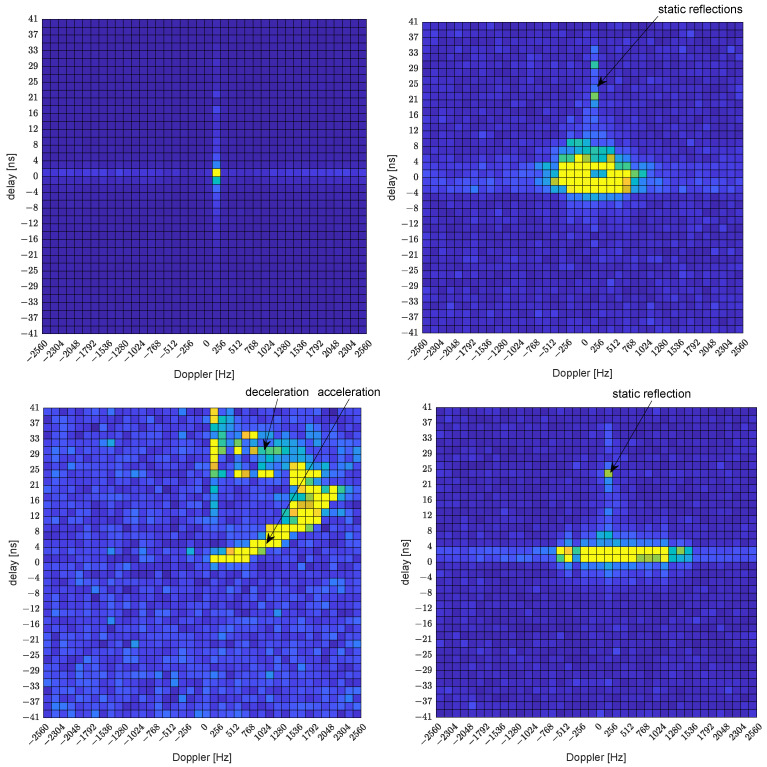
Max-hold plots of delay-Doppler profiles for example person activities—static person (**top left**), randomly walking person (**top right**), sprinting person (**bottom left**), and dancing person (**bottom right**).

In a way similar to in [7], the information about the sensed environment is first presented in Figure 9 in the form of max-hold delay-Doppler plots for the four scenarios described above. Even in the case of the static person, slight leakage of the channel tap due to the delay-Doppler grid mismatch is visible. In all three moving scenarios, the reflections from static objects at similar distances from the sensing receiver can be identified. Note that a delay of ≈ 29 ns is in good agreement with a monitored area width of 4.5 m, see Figure 8. This static reflection usually happens in the situation when the person is out of the range of the used horn antennas, e.g., at the beginning or end of the measurements. In the scenario of the random walk of the person in the monitored area (scenario 2), we can notice that the movement of the person was permanent, as the grid element corresponding to zero velocity has negligible magnitude (not equal to zero as a result of leakage due to the grid mismatch). In the plot representing the running person (scenario 3), we can easily identify three phases of movement—acceleration, running with a constant speed, and deceleration (the person had to stop prior to reaching the room wall). The Delay-Doppler plot for the last scenario—the dancing person (scenario 4)—contains important Doppler shifts due to the substantial movements of the body and the limbs, but the distance from the sensing receiver remains limited.

### 5.2. Performance of CFAR and Persistent Homology Detectors on Measured Data

**Figure 10:**
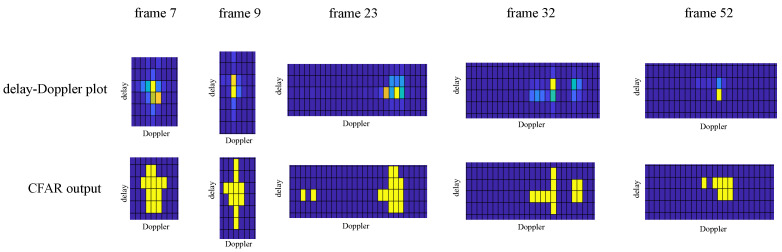
Conventional CFAR in the presence of delay-Doppler grid mismatch for selected OTFS frames.

Firstly, we have been interested in the behavior of the conventional CFAR detector on the real data, with the non-integer ratio between the real delay and Doppler shift of the reflected components and their OTFS grid counterparts. Example results are shown in Figure 10. Through these results, we point out that the conventional CFAR detector may lead to the emergence of a number of falsely detected targets. For frames 7 and 9, all the points in the grid above the threshold are neighboring, and the detector identifies a single target. This is not the case for the three remaining example frames (23,32,52) for which the two (frames 32 and 52) or even three (frame 23) isolated objects are identified. Note that for frame number 32, the persistent homology-based detector also identifies two distinct targets.

**Figure 11:**
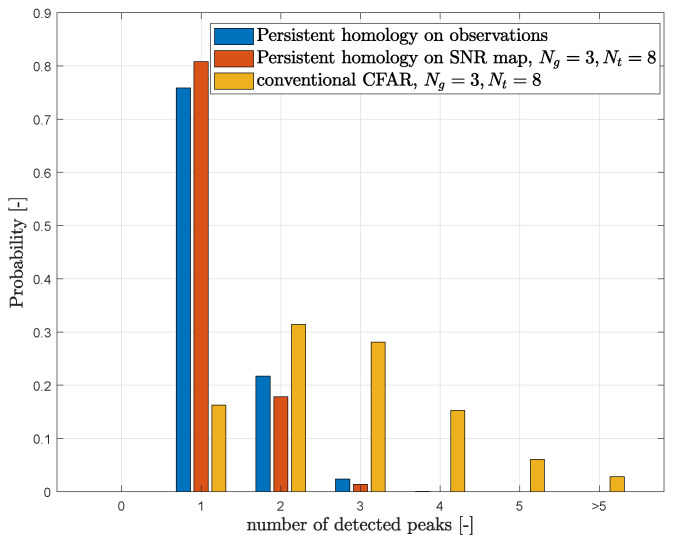

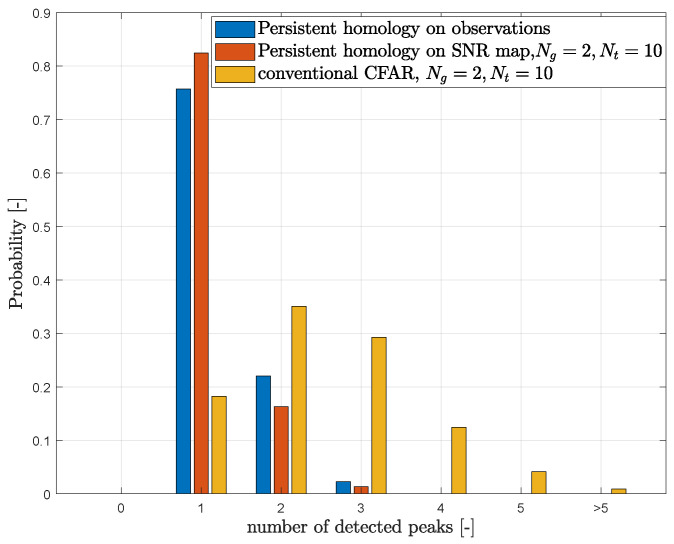
Comparison of various target detection techniques for two sets of training and guard cell settings of CFAR detector. Results were obtained by averaging 12 realizations of OTFS transmission, with ≈300 delay-Doppler images for each realization. During all experiments, a single person was present in the monitored environment, i.e., the expected number of detected peaks is equal to 1. In less than 10% of images, the reflection from a static object is present (distance of approx. 4.5 m, see Figure 9).

A numerical comparison of the overall performance of three used target detectors is provided in Figure 11, which shows the probability of the number of detected targets (isolated peaks in delay-Doppler images). These results have been obtained by averaging over 12 distinct realizations of single-person movement in the monitored area. Each realization contains about 300 OTFS slots on average. The results confirm a large number of falsely detected targets in the case of the basic CFAR algorithm for both configurations of guard and training cells. The use of the two remaining detectors employing the persistent homology leads to a much lower number of false targets, with the persistent homology method on the SNR map providing only a slightly higher probability of the detection of a single target (one human presence).

**Figure 12:**
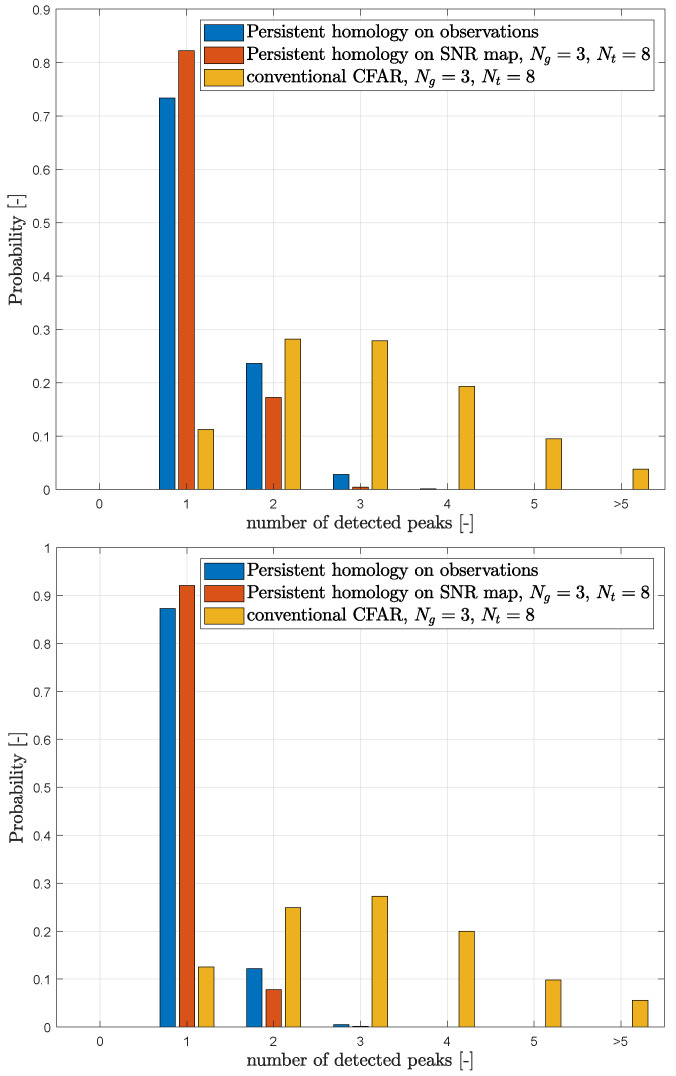

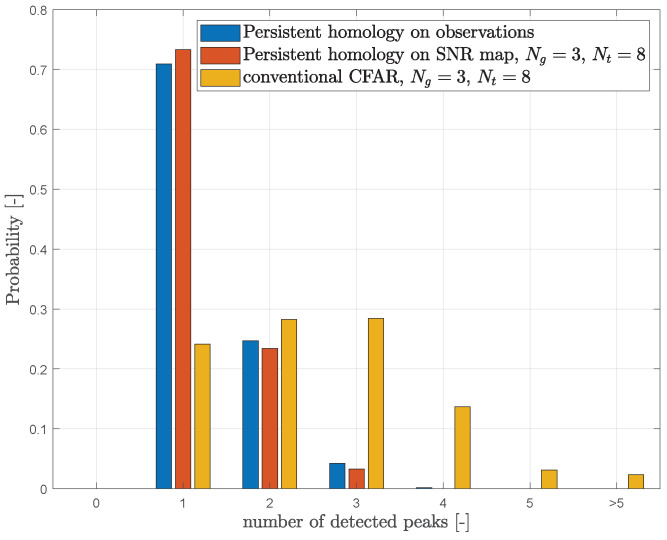
Analysis of target detector performance for various activities: walking (**top**), jumping (**middle**), and running (**bottom**).

To provide better insight into the performance of the three investigated detectors, Figure 12 compares the peak detection probability for three different kinds of activities—a slow walk, jumping, and a fast sprint of a person—that correspond to three distinct delay-Doppler variations—the slow changes in both delay and Doppler domains, the fast changes in the Doppler domain only, and fast changes in both delay and Doppler domains. The detectors based on the persistent homology principle achieve the best performance in the case of changes in the Doppler domain only (jumping of the person at a constant distance from the receiver). On the contrary, the worst performance is achieved for very fast changes in both delay and Doppler domains (sprinting person).

## 6. Conclusions

In this paper, we presented the principle of a persistent homology approach for the detection of the targets (more specifically, a person) from the 2D delay-Doppler images. We presented our unique millimeter-wave measurement test-bed, which is able to continuously transmit and receive communication signals in an unlicensed 60 GHz band. This setup was used to transmit the OTFS signals with a bandwidth of ≈ 500 MHz, and the received signals reflected from the person in the environment were used to generate the delay-Doppler data for detection. We compared the performance of the proposed detector with a detector based on the standard CFAR approach. In the selected scenario of various movements of one person in real conditions, the results show the superiority of the persistent homology over the conventional CFAR detector. So far, we have used a simple implementation of the persistent homology detector, with higher computational demands than required by the conventional CFAR detector. An in-depth optimization of the detector structure and parameters would be an interesting topic for future work. An important research question is a search for the best pre-processing of data fed to the input of the detector. We presented our first attempts in this direction by showing the detector performance on two types of input data—the rough observations in the delay-Doppler domain, and the SNR map computed from the delay-Doppler domain images.

## Figures and Tables

**Table 1:** OTFS system parameters.

*M*, number of subcarriers [-]	1900
$M_{0}$, number of zero subcarriers [-]	100
*N*, number of symbols in OTFS slot [-]	2000
$\Delta_{d}$, delay resolution	2.1 ns
$\Delta_{D}$, Doppler resolution	128 Hz
Number of pilot guards in delay domain [-]	48
Number of pilot guards in Doppler domain [-]	48
Sampling frequency	512 MHz
Signal bandwidth	≈490 MHz
Data symbols	4-QAM, uncoded
Communication data rate	≈920 Mbit/s

**Table 2:** Parameters and time complexity per one delay-Doppler image for three investigated detectors. N/A stands for “Not applicable.”

$N_{g}$ Cells	$N_{t}$ Cells	$P_{\mathrm{fa}}$	z-Score Threshold	Time Complexity [ms]
**Conventional CFAR**				
3	8	$1\times 10^{-6}$	N/A	10.0
2	10	$1\times 10^{-6}$	N/A	9.9
**Persistent homology applied on observations**				
N/A	N/A	N/A	6	264.6
N/A	N/A	N/A	6	243.2
**Persistent homology applied on SNR map**				
3	8	N/A	6	269.5
2	10	N/A	6	249.8

## Data Availability

The complete data set is not publicly available due to the data size (28 GB for 14 s of measurements). Sample delay-Doppler matrices are available on request.

## Abbreviations

The following abbreviations are used in this manuscript:

OTFS	Orthogonal Time Frequency Space
ISaC	Integrated Sensing and Communications
CFAR	Constant False Alarm Rate
6G	Sixth-Generation
UWB	Ultra Wide Band
FMCW	Frequency Modulated Continuous Wave
OFDM	Orthogonal Frequency Division Multiplex
WLAN	Wireless Local Area Network
IEEE	Institute of Electrical and Electronics Engineers
SDR	Software Defined Radio
2D	Two-dimensional
QAM	Quadrature Amplitude Modulation
ISFFT	Inverse Symplectic Finite Fourier Transform
CP	Cyclic Prefix
CSI	Channel State Information
BER	Bit Error Rate
TDA	Topological Data Analysis
DAC	Digital to Analog Converter
ADC	Analog to Digital Converter
SNR	Signal to Noise Ratio
CPU	Central Processor Unit
